# FedHSFV: Federated Learning for Finger Vein Recognition via Hierarchical Decoupling and Subspace Metric

**DOI:** 10.3390/s26134322

**Published:** 2026-07-07

**Authors:** Ximing Zhou, Yuhan Wang, Jiajun Cui, Jian Guo, Hengyi Ren

**Affiliations:** 1 School of Computer Science, Nanjing University of Posts and Telecommunications, Nanjing 210023, China; b23040522@njupt.edu.cn (X.Z.);; 2 Jiangsu Provincial Key Laboratory of Internet of Things Intelligent Perception and Computing, Nanjing University of Posts and Telecommunications, Nanjing 210023, China; 3 College of Information Science and Technology & Artificial Intelligence, Nanjing Forestry University, Nanjing 210037, China

**Keywords:** finger vein recognition, personalized federated learning, hierarchical decoupling, subspace analysis

## Abstract

Finger vein recognition (FVR) has significant potential in biometrics due to its high accuracy and intrinsic liveness detection capabilities. However, the increasingly stringent privacy regulations have presented severe data security challenges for traditional centralized training. While federated learning (FL) mitigates these privacy concerns through a decentralized training paradigm, conventional FL algorithms that seek a single global model experience significant performance degradation on non-independent and identically distributed (Non-IID) data in real-world cross-institutional deployments. This degradation stems primarily from a dual-heterogeneity issue that involves domain shift caused by hardware discrepancies across acquisition devices, and label skew resulting from nonoverlapping user identities. To address this dual-heterogeneity challenge, we propose a personalized federated learning framework driven by hierarchical parameter decoupling and subspace metric. First, we designed a hierarchical parameter decoupling architecture. Macroscopically, the architecture retains the classifier locally to isolate label heterogeneity; microscopically, it introduces an additive parameter decomposition that decouples the feature extractor on a global full-rank basis (to capture domain-invariant semantics, namely, the shared physiological vein topologies) and a local low-rank adapter (that accommodates device-specific characteristics, such as hardware-induced noise and illumination discrepancies). Furthermore, we propose a subspace similarity matching strategy based on principal angles on the Grassmann manifold. By exploiting the geometric properties of low-rank projection matrices, this strategy accurately quantifies the underlying distribution discrepancies among clients to guide personalized weighted aggregation. Extensive experiments on six public finger vein datasets demonstrate that the proposed framework significantly improves the overall recognition performance and mitigates performance degradation caused by data heterogeneity.

## 1. Introduction

Finger vein recognition (FVR), as an emerging biometric authentication technology, has become a crucial research direction in the field of identity security, due to its non-intrusiveness, anti-spoofing, and intrinsic liveness detection capabilities [1]. In recent years, deep learning has achieved substantial progress in extracting vein texture features [2,3,4]; however, its robustness heavily relies on massive amounts of centralized training data [5]. In real-world deployments, due to the increasingly stringent privacy protection regulations [6,7,8], sensitive biometric data across different institutions cannot be shared, forming strict “data silos” that severely hinder model generalization. Federated learning (FL), as a privacy-preserving decentralized training paradigm, provides an effective pathway to break down these silos.

**Figure 1:**
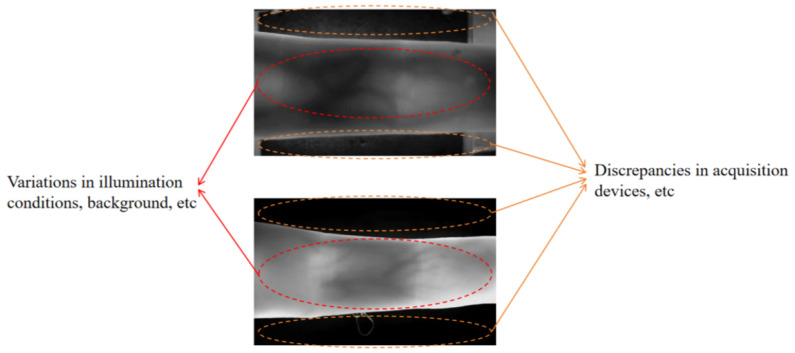
Heterogeneity dilemmas in cross-institutional finger vein recognition, illustrating the challenges of domain shift and label skew.

Despite the significant potential of FL in the FVR domain, it faces a highly challenging “dual-heterogeneity” issue in practical cross-institutional deployments. Specifically, this problem involves (i) domain shift caused by variations in acquisition devices and imaging environments and (ii) label skew resulting from nonoverlapping user populations across institutions. As intuitively illustrated in Figure 1, finger vein images captured by different clients exhibit substantial visual discrepancies in illumination conditions, background noise, and device-specific edge features. When confronting such strong non-independent and identically distributed (Non-IID) characteristics, the traditional Federated Averaging (FedAvg) algorithm often suffers from severe feature drift and performance degradation. To address this challenge, the existing research has largely pivoted toward personalized federated learning (PFL) mechanisms [9,10,11]. However, these methods typically apply personalization to all of the entire model parameters or perform only coarse-grained layer isolation, which not only incurs prohibitive communication overhead but also easily causes the model to lose the delicate balance between “overfitting to local noise” and “catastrophic forgetting of global consensus”.

To effectively address the aforementioned heterogeneity challenges, it is imperative to re-evaluate the feature representations and parameter aggregation metrics in FVR data. On the one hand, the client-specific “background noise” (e.g., illumination variations and device sensor characteristics) is deeply coupled with the universal core “vein texture”. If not properly disentangled, direct aggregation will inevitably degrades the generalization performance of the global model; moreover, the inconsistency of the label spaces mandates effective isolation at the classifier level. On the other hand, the existing parameter aggregation strategies predominantly rely on the Euclidean distance in the parameter space or simple scalar weight calculations [12,13,14]. Such metric approaches severely overlook the inherent manifold geometric structure of high-dimensional weight matrices in neural networks, making it difficult to precisely quantify and align the essential discrepancies of cross-domain data distributions in highly heterogeneous scenarios. Therefore, this paper introduces the concepts of hierarchical decoupling [15,16] and subspace geometric metric [17] to achieve precise feature matching across domains while stripping away domain-specific noise.

Motivated by these observations, we propose a personalized federated learning framework based on hierarchical parameter decoupling and subspace metric. Unlike the existing works [18,19], which implicitly address heterogeneity solely at the model aggregation level, we explicitly decompose features from a physical perspective and partition the training process into two stages: consensus first, followed by personalization. With respect to the model architecture, we explicitly decouple the feature extractor and the classifier at the macroscopic level, strictly retaining the classifier locally to mitigate the negative transfer induced by global label skew. Microscopically, inspired by additive adaptation [20,21,22], we perform an additive decomposition on the feature extractor, reconstructing it into a shared “global full-rank basis” and a private “local low-rank adapter”. This design is highly tailored to the physical attributes of finger vein recognition, explicitly disentangling low-rank physical interferences, such as near-infrared scattering and uneven illumination, from the high-rank vein topological semantics that determine identity. In terms of the training mechanism, since the noise distributions across different devices reside in distinct low-dimensional subspaces, traditional Euclidean distance metrics are ineffective. Therefore, we introduce principal angles on the Grassmann manifold [23] as the similarity metric. This metric effectively characterizes the geometric relationships between subspaces and perfectly accommodates the physical reality of FVR in that noise subspaces exhibit a low-dimensional manifold structure, thereby enabling the construction of customized, secure collaborative aggregation weights for each client.

The main contributions of this paper are summarized as follows:

(1) A novel two-stage personalized federated learning paradigm is proposed. It integrates generalization and personalization objectives—the first stage strengthens global consensus, while the second focuses on personalized fine-tuning—providing a systematic solution for cross-domain FVR.
(2) We design a hierarchical parameter decoupling architecture that integrates macroscopic classifier isolation with microscopic additive parameter decomposition. This approach effectively resolves the dilemmas of label skew and domain shift without increasing the computational burden on the backbone network.
(3) A subspace similarity metric based on principal angles is introduced in this framework. By deeply mining the geometric manifold features of the low-rank adapter matrices, it overcomes the metric limitations of the traditional Euclidean distance, enabling precise quantification of data distribution discrepancies and fine-grained weight allocation in extremely heterogeneous environments.
(4) In this study, extensive empirical evaluations were conducted. Comprehensive comparative experiments on six highly heterogeneous public FVR datasets demonstrate that the proposed framework not only effectively overcomes the performance degradation induced by data heterogeneity but also significantly outperforms mainstream FL methods in terms of their core recognition metrics and cross-domain stability.

## 2. Related Work

### 2.1. Finger Vein Recognition

As a high-security biometric recognition technology, finger vein recognition uses near-infrared imaging to capture subcutaneous vascular networks for identity authentication. The early feature extraction algorithms primarily relied on hand-crafted descriptors, such as methods based on maximum curvature points [24], repeated line tracking [25], and local texture features [26]. While these traditional methods can achieve acceptable recognition accuracy in controlled environments, their inherent reliance on manually designed feature templates makes them highly subjective. Consequently, they often exhibit poor robustness when processing low-quality images or when handling complex illumination variations.

In recent years, driven by the rapid development of deep convolutional neural networks (CNNs), data-driven deep learning methods [27,28] have gradually replaced traditional approaches, emerging as the mainstream paradigm for FVR. Deep models can automatically extract more comprehensive and discriminative high-dimensional features in an end-to-end manner. For example, Das et al. [29] were among the first to adopt a multilayer CNN architecture to process finger vein images, significantly reducing the system’s equal error rate. Subsequent research introduced more complex hybrid architectures; for instance, Abbas et al. [30] combined CNNs with long short-term memory (LSTM) networks to capture spatial sequence features, and Ma et al. [31] utilized a dual attention mechanism to focus on regions of interest within vein textures, providing new insights for feature extraction. Overall, deep learning methods clearly outperform traditional methods that require manual feature extraction. However, the superior performance of deep models relies heavily on large-scale, diverse, and centralized training data. In real-world application scenarios, constrained by increasingly stringent data privacy regulations and high collection costs, it is difficult for a single institution to independently acquire massive datasets. This ubiquitous “data silo” phenomenon severely restricts the generalization capability of models in practical cross-domain deployments.

### 2.2. Finger Vein Recognition Based on Federated Learning

To overcome the aforementioned “data silo” dilemma and enhance model generalization in cross-domain deployments, federated learning, which serves as a privacy-preserving distributed machine learning framework, has been widely adopted in the FVR field [32,33,34]. Through a decentralized collaborative training mechanism, FL allows multiple institutions to share only locally trained model parameters without uploading raw, sensitive biometric data. This approach not only complies with the stringent data security regulations but also provides an ideal pathway for the collaborative optimization of cross-domain FVR models.

In practical cross-domain deployments, the “domain shift” caused by device discrepancies and the “label skew” that results from mutually exclusive user populations introduce severe Non-IID challenges. Consequently, recent research has focused largely on and pivoted towards personalized federated learning (PFL). Specifically, for finger vein scenarios, PaFedFV [10] introduced an asynchronous normalization mechanism to enhance device adaptability; FedPPA [19] attempted to mitigate catastrophic forgetting through progressive parameter alignment; and FedPSFV [35] approached the problem by utilizing prototype sharing to constrain features at a global level. However, these cutting-edge strategies—regardless of whether they are based on parameter fine-tuning or coarse-grained constraints—often ignore the inherent manifold geometric structure of high-dimensional weight matrices in neural networks. Thus, fundamentally and effectively disentangling the highly coupled “universal semantics” and “device-specific physical noise” within deep features remains difficult. Although the latest FL studies [17,23] have begun exploring collaborative schemes based on the subspace and manifold geometry to overcome the limitations of traditional Euclidean metrics, these methods have yet to be effectively integrated into deep feature decoupling tasks. Without prior isolation of the underlying physical noise, performing manifold metrics directly within a global subspace still incurs severe feature bias.

To address these limitations, this paper proposes a novel PFL framework named FedHSFV, which is driven by subspace metric and hierarchical parameter decoupling. This framework introduces macroscopic and microscopic hierarchical parameter decoupling to effectively eliminate device-level physical noise. Furthermore, it departs from traditional Euclidean metrics, opting instead to leverage principal angles on the Grassmann manifold to accurately quantify subspace similarities among clients. This geometrically aware strategy guides more robust personalized weighted aggregation, achieving a simultaneous enhancement in both cross-domain model generalization and individual personalization capabilities.

## 3. Methodology

To address the significant data distribution discrepancies and label misalignment dilemmas in cross-domain finger vein recognition, this section elaborates on the underlying logic and core algorithmic mechanisms of the proposed FedHSFV framework. This system integrates hierarchical parameter decoupling with subspace geometric metrics, aiming to achieve a dynamic balance between global generalization and local personalization in heterogeneous environments.

### 3.1. Problem Formulation

In real-world finger vein recognition applications, sensitive biometric data are typically isolated within different institutional servers. To fully replicate this Non-IID federated learning deployment scenario, in this study, we constructed a federated learning environment comprising *K* heterogeneous clients.

Let $C=\{C_{1},\ldots ,C_{K}\}$ denote the set of clients. Each client k∈{1,…,K} holds a private local dataset $D_{k}={\left\{x_{i}^{\left(k\right)},y_{i}^{\left(k\right)}\right\}}_{i=1}^{N_{k}}$, where the feature vector $x_{i}^{\left(k\right)}\in X$ represents the input finger vein image and $y_{i}^{\left(k\right)}\in Y_{k}$ denotes the corresponding ground-truth label. Here, X and $Y_{k}$ represent the global feature space and the local label space of client *k*, respectively. The size of each private dataset is $N_{k}$, and the total size of all of the client datasets is $N=\sum_{k=1}^{K}N_{k}$.

Since these data are distributed across different institutions, the system faces a dual-heterogeneity challenge:

(1) Domain Shift: Discrepancies in the sensor parameters, illumination conditions, and imaging principles among different devices lead to severe inconsistencies in the marginal feature distributions.
(2) Label Skew: The user identities enrolled by different institutions are mutually exclusive, resulting in a strictly nonoverlapping global label space.

Under the aforementioned settings, in addition to the *K* clients, a central server is deployed to coordinate the global training process. The ultimate goal of our proposed personalized federated learning framework is to obtain a set of optimal personalized model parameters $\Theta^{*}=\left\{\theta_{1}^{*},\ldots ,\theta_{K}^{*}\right\}$ through secure collaboration, which minimizes the weighted sum of the local empirical risk across all clients. Specifically, the complete personalized parameters $\theta_{k}^{*}$ for each client *k* are rigorously defined as $\theta_{k}^{*}=\left\{W_{\mathrm{global}}^{(k)*},B^{(k)*},A^{(k)*},\phi_{k}^{*}\right\}$. Here, $W_{\mathrm{global}}^{(k)*}$ denotes the full-rank feature basis participating in the global consensus aggregation during the first phase, and $B^{(k)*}$ serves as the public corrector involved in the server-side subspace metric computation during the second phase. Conversely, $\left\{A^{(k)*},\phi_{k}^{*}\right\}$ represent the private perceiver and classifier parameters, respectively, which are completely frozen and strictly retained locally to isolate device-specific physical noise and label skew. This optimization objective is formulated as defined in Equation (1):

(1) $$\Theta^{*}=\arg\min_{\left\{\theta_{1},\ldots ,\theta_{K}\right\}}\sum_{k=1}^{K}\frac{N_{k}}{N}F_{k}\left(\theta_{k}\right)$$

where $F_{k}\left(\theta_{k}\right)$ represents the local empirical loss function of client *k* on its private dataset $D_{k}$, which is specifically defined as shown in Equation (2):

(2) $$F_{k}\left(\theta_{k}\right)=\frac{1}{N_{k}}\sum_{i=1}^{N_{k}}L_{k}\left(\theta_{k};x_{i}^{\left(k\right)},y_{i}^{\left(k\right)}\right)$$

where $\theta_{k}$ denotes the personalized network model parameters assigned to client *k*, and $L_{k}$ is the loss function used to evaluate the prediction error for a single sample on client *k*.

### 3.2. FedHSFV Framework

**Figure 2:**
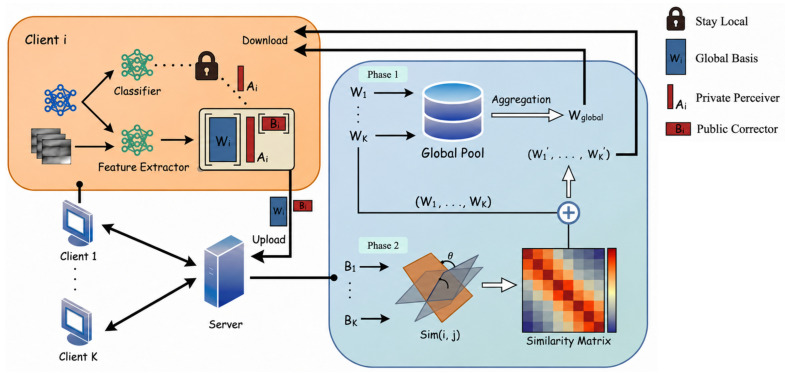
Overview of the proposed FedHSFV framework.

To effectively address the dual data heterogeneity dilemmas in cross-domain finger vein deployments, this work proposes a two-stage personalized federated learning framework driven by subspace metric. From a holistic system perspective, the operational logic of this framework intertwines the local hierarchical decoupling mechanism on the client side with the global collaborative mechanism on the server, which is based on manifold metrics. As illustrated in Figure 2, the overall federated optimization process is designed into two progressive stages. In the first phase, clients independently optimize and extract cross-domain universal features on the basis of a local hierarchical decoupling architecture, which are then securely aggregated by the central server to achieve the preliminary alignment of global consensus. In the second phase, the server performs manifold metrics and directional weighting based on the client-specific features, and then distributes customized parameters to guide each client in completing the local optimization update of their personalized models.

Phase 1: Global Consensus Warmup. All clients locally deploy a hierarchical parameter decoupling architecture: macroscopically, private classifiers are strictly isolated to circumvent cross-domain label skew; microscopically, an additive decomposition is applied to the feature extractor, reconstructing it into a shared “global full-rank basis” and a specific “local low-rank adapter”. After each client independently iterates this decoupled model on private data, the central server collects and securely aggregates only the “global bases” from all nodes by using the Federated Averaging (FedAvg) strategy. Under the premise of protecting data privacy, this phase efficiently accomplishes the preliminary alignment of cross-domain shared semantics.

Phase 2: Subspace Matching and Personalized Synergy. Each client uploads its “low-rank adapter”–which reflects the specific distribution of local data—as a subspace signature. Upon receiving these signatures, the central server calculates the principal angles between the signature matrices on the Grassmann manifold to construct a client similarity matrix. Finally, relying on this matrix, the server calculates directed aggregation weights for each client and distributes customized network parameters, guiding the clients to fine-tune high-performance personalized models locally.

The detailed algorithmic flow is presented in Algorithm 1.
**Algorithm 1** FedHSFV: Two-Phase Collaborative Training**Require:** Total communication rounds *T*, Phase 1 rounds $T_{1}$, local epochs *E*.**Ensure:** Personalized feature extractors $W^{\left(k\right)}=W_{global}^{\left(k\right)}+B^{\left(k\right)}A^{\left(k\right)}$.  1:**Initialize:** Server basis $W_{global}^{0}$; Client adapters $A^{\left(k\right)}$, correctors $B^{\left(k\right)}$, classifiers $\phi_{k}$, and class centers $c^{\left(k\right)}$.  2:Phase 1: Global Consensus Warm-up  3:**for**$\;t=0,\ldots ,T_{1}-1\;$**do**  4:   **for** each client k∈{1,…,K} in parallel **do**  5:     Construct $W^{\left(k\right)}\leftarrow W_{global}^{t}+B^{\left(k\right)}A^{\left(k\right)}$  6:     Update local parameters via *E* epochs of SGD to minimize $L_{k}=L_{CE}+\lambda L_{Center}$  7:     Upload $W_{global}^{(k),t+1}$ to server $\left\{\mathrm{Keep}\;A^{\left(k\right)},\phi_{k},c^{\left(k\right)}\mathrm{strictly}\;\mathrm{local}\right\}$  8:   **end for**  9:   Server broadcasts $W_{global}^{t+1}\leftarrow \frac{1}{K}\sum_{k=1}^{K}W_{global}^{(k),t+1}\;\{\mathrm{FedAvg}\}$10:**end for**11:Phase 2: Subspace Matching and Personalized Synergy12:Server collects ${\left\{B^{\left(k\right)}\right\}}_{k=1}^{K}$, computes orthogonal bases via QR decomposition, and calculates subspace similarity matrix *S* via principal angles.13:Server computes and freezes customized weights $\alpha_{i,j}\propto \exp\left(S_{i,j}/\tau \right)$.14:**for**$\;t=T_{1},\ldots ,T-1\;$**do**15:   **for** each client k∈{1,…,K} in parallel **do**16:     Construct $W^{\left(k\right)}$ and update local parameters via SGD to minimize $L_{k}$.17:     Upload $W_{global}^{(k),t+1}$ to server.18:   **end for**19:   **for** each client i∈{1,…,K} **do**20:     Server computes customized basis: $W_{global}^{(i),t+1}\leftarrow \sum_{j=1}^{K}\alpha_{i,j}W_{global}^{(j),t+1}$21:     Send customized $W_{global}^{(i),t+1}$ to client *i*.22:   **end for**23:**end for**

### 3.3. Phase 1 of FedHSFV

To effectively isolate device-specific physical noise and adapt to the mutually exclusive label spaces across institutions, this paper proposes a hierarchical decoupling paradigm that intertwines the macroscopic and microscopic levels within the local client model. This architecture explicitly delineates the update boundaries of different network modules, achieving efficient knowledge disentanglement without introducing additional computational burden.

Specifically, in a single round of local training, the complete neural network of client *k* is first explicitly decoupled at the macroscopic level into two independent modules: a feature extractor $F(\cdot ;W^{\left(k\right)})$, which is responsible for feature representation, and a classifier $C(\cdot ;\phi_{k})$, which is responsible for label mapping. Given an input finger vein image from the private dataset, the data first pass through the feature extractor to extract the deep vein features.

To overcome the domain shift caused by the physical properties of different acquisition devices in real time during the feature extraction process, the weights within the feature extraction network undergo additive low-rank decomposition at the microscopic level. Specifically, this decomposition is exclusively deployed on all of the convolutional layers within the feature extractor backbone, whereas the nonparametric pooling or normalization layers remain unaltered, thereby precisely isolating the device-specific noise within the spatial receptive field while strictly controlling the computational overhead. When the image feature flows through any convolutional network layer with an input feature dimension of $d_{in}$ and an output feature dimension of $d_{out}$, its local network weight matrix $W^{\left(k\right)}$ is additively decomposed into a shared full-rank global basis $W_{global}$ and a domain-specific local adaptation term $\Delta W^{\left(k\right)}$. Here, $W_{global}$ is responsible for capturing cross-domain domain-invariant vein texture features. To efficiently fit the local domain features and strictly limit the communication overhead, we impose a low-rank constraint on $\Delta W^{\left(k\right)}$, further decomposing it into a private perceiver $A^{\left(k\right)}\in R^{r\times d_{in}}$ and a public corrector $B^{\left(k\right)}\in R^{d_{out}\times r}$, where the hyperparameter *r* is the constraint rank satisfying $r\ll \min(d_{in},d_{out})$. Since the domain shift induced by the physical discrepancies of the acquisition devices typically manifests as systematic biases on a low-dimensional manifold, an *r* value significantly smaller than the feature dimensions naturally presents a robust information bottleneck. This bottleneck compels the low-rank adapter to selectively filter and fit only the most prominent variance-dominant features (i.e., device-specific noise) within the local domain data, effectively preventing local overfitting caused by an excessively high dimension and underfitting of the domain shift caused by an overly small dimension. The aforementioned mapping calculation strictly follows Equation (3):

(3) $$W^{\left(k\right)}=W_{global}+\Delta W^{\left(k\right)}=W_{global}+B^{\left(k\right)}A^{\left(k\right)}$$

In this microscopic decoupling architecture, $W_{global}$ retains the full-rank representational capacity, which is essential for encoding the complex, high-frequency finger vein topological structures shared across all clients. By projecting the input features into a low-dimensional subspace, $A^{\left(k\right)}$ explicitly strips away redundant identity semantics to obtain the most salient, variance-dominant physical noise specific to the local device. Subsequently, $B^{\left(k\right)}$ maps these low-dimensional noise features back into the high-dimensional output space. During this process, the universal features extracted by the global basis $W_{global}$ are superimposed with the specific noise captured and inverted by $A^{\left(k\right)}B^{\left(k\right)}$, effectively neutralizing the inherent physical interference of the device. As the data flow through the entire feature extraction network, highly purified deep vein features $f_{i}$ are ultimately generated.

The generated deep features $f_{i}$ are subsequently input into the macroscopically decoupled classifier $C(\cdot ;\phi_{k})$, where they are mapped to the unique label space of the current client by using the private parameters $\phi_{k}$ to generate category prediction probabilities. Regarding the collaborative training strategy, the global full-rank basis and the local low-rank adapter adopt an end-to-end joint optimization paradigm. During the model initialization phase, $W_{global}$ and $A^{\left(k\right)}$ utilize standard random initialization, whereas the public corrector $B^{\left(k\right)}$ is initialized to zero. These settings ensure that the supplementary low-rank branch is entirely zero at the initial state, preventing initial random noise from disrupting the global feature extraction process. The system calculates the local joint loss based on the prediction probabilities and executes backpropagation to complete the parameter updates. The specific construction of the joint loss function and its physical significance are described in Section 3.5.

The decoupled network parameters are then directionally distributed according to their carried information attributes: the private perceiver $A^{\left(k\right)}$, which contains the locally sensitive distributions and the classifier $\phi_{k}$, which handles local label skew are permanently frozen and retained locally. The full-rank global basis $W_{global}$, having captured the cross-domain universal features, is submitted to the central server for preliminary consensus alignment via Federated Averaging. Moreover, the public corrector $B^{\left(k\right)}$, which abstracts the geometric characteristics of the local domain, is uploaded to the server for the subspace similarity measurement and personalized synergy in the second phase.

### 3.4. Phase 2 of FedHSFV

This section focuses on the core algorithmic mechanism at the central server. The central motivation is to leverage the public correctors uploaded by the clients to measure the interdomain discrepancies within the manifold space, thereby guiding the secure aggregation of knowledge. To achieve this objective, the operations on the server side are divided into two sequential steps: “similarity quantification” and “directional weighting”.

Quantifying the cross-domain subspace similarity: Traditional federated learning predominantly relies on the Euclidean distance in the parameter space to measure model discrepancies. However, this metric is highly susceptible to the inherent rotational and scaling ambiguities present in matrix factorization, leading to suboptimal robustness. To accurately capture the essential geometric structure of the interdomain differences, in this work, the uploaded low-rank adapter matrices $B^{\left(k\right)}$ are modelled as subspace generators on the Grassmann manifold $G(r,d_{out})$. Mathematically, the Grassmann manifold $G(r,d_{out})$ is strictly defined as the set of all *r*-dimensional linear subspaces within a $d_{out}$-dimensional real vector space. The client-specific feature shifts are inherently embedded within the subspace spanned by the column vectors of $B^{\left(k\right)}$.

To eliminate the scaling ambiguity caused by the selection of specific basis vectors, for any two clients *i* and *j*, we first perform a thin QR decomposition [36] on the uploaded matrices $B^{\left(i\right)}$ and $B^{\left(j\right)}$. This process yields their unique orthogonal basis matrices $Q_{i}$ and $Q_{j}$, as formulated in Equation (4):

(4) $$B^{\left(i\right)}=Q_{i}R_{i},\;B^{\left(j\right)}=Q_{j}R_{j}$$

where $R_{i}$ and $R_{j}$ are non-singular upper triangular matrices that contain the scaling and shear information of the original basis vectors. Since the geometric manifold properties of a subspace are entirely determined by its orthogonal basis Q, discarding R in subsequent metrics mathematically isolates the subspace from the numerical scaling ambiguities induced by different local training paths.

Subsequently, according to subspace geometry theory, the structural alignment between two subspaces can be rigorously quantified by their principal angles. The cosines of these principal angles are mathematically equivalent to the singular values of the orthogonal projection matrix $Q_{i}^{T}Q_{j}$. Therefore, the server executes singular value decomposition (SVD) [37] on this inner product matrix, as shown in Equation (5):

(5) $$U\Sigma V^{T}=\mathrm{SVD}\left(Q_{i}^{T}Q_{j}\right)$$

Here, the singular values $\sigma_{n}$ within the diagonal matrix $\Sigma =\mathrm{diag}(\sigma_{1},\ldots ,\sigma_{r})$ correspond precisely to the cosines of the *n*-th principal angles $\theta_{n}$ between the two subspaces (i.e., $\sigma_{n}=\cos\theta_{n}$). The final subspace similarity score is defined as the mean of these *r* principal angle cosines, as shown in Equation (6):

(6) $$Sim\left(i,j\right)=\frac{1}{r}\sum_{n=1}^{r}\sigma_{n},\;Sim\left(i,j\right)\in \left[0,1\right]$$

This manifold metric mechanism theoretically possesses rotational invariance, thus seamlessly shielding the aggregation process from the numerical perturbations caused by different local initializations or optimization paths, and accurately captures the structural alignment state of the optimization directions across different devices.

Executing personalized weighted synergy: After obtaining all of the similarity scores, the server uses this matrix to guide personalized network aggregation. To further widen the weight gap between strongly correlated and irrelevant clients, the server introduces a temperature coefficient τ [38] and calculates the directed aggregation weight $\alpha_{i,j}$ from collaborative client *j* to target client *i* via the softmax function, as defined in Equation (7):

(7) $$\alpha_{i,j}=\frac{\exp(Sim(i,j)/\tau )}{\sum_{m=1}^{K}\exp\left(Sim\left(i,m\right)/\tau \right)}$$

Let the current round in the personalized synergy phase be t+1 (where *t* is the index of the completed global communication rounds from the previous phase). Based on these weights, the server dynamically generates a customized next-round global basis $W_{global}^{(i),t+1}$ for each target client *i*, as shown in Equation (8):

(8) $$W_{global}^{(i),t+1}=\sum_{j=1}^{K}\alpha_{i,j}W_{global}^{(j),t+1}$$

Upon completing the customized aggregation, the server directionally distributes $W_{global}^{(i),t+1}$ to client *i*. The client receives it and recombines it with the locally frozen private perceiver $A^{\left(i\right)}$ and classifier $\phi_{i}$, reconstructing a complete personalized model for subsequent fine-tuning and inference evaluation. This closed-loop mechanism introduces positive transfer gains from similar domains while fundamentally resisting the negative interference and catastrophic forgetting caused by heterogeneous distributions.

### 3.5. Local Joint Optimization Objective

At the model optimization level, finger vein recognition fundamentally adheres to an open-set verification protocol, meaning that the user identities encountered by the model during the actual inference and deployment stages are completely unseen during local training. This rigorous setting mandates that the feature extraction network not only distinguishes known classes in the training set but also learns highly generalizable discriminative feature representations.

Although the traditional cross-entropy loss can effectively increase the interclass distances, prompting samples to be linearly separable in the feature space, it lacks explicit constraints on the intraclass distribution. This limitation often leads to scattered distributions of extracted intraclass features in space. When faced with heterogeneous noise from cross-domain devices (e.g., uneven illumination and scattering differences), such loose feature clustering makes the features highly susceptible to severe drift, crossing decision boundaries and causing system misrecognition. To overcome this deficiency, this paper introduces centre loss [39] into the local client training. By dynamically maintaining a learnable class centre for each identity category in the deep feature space and actively penalizing the Euclidean distance between deep features and their corresponding class centres, this loss function explicitly minimizes the intraclass variance, enforcing high intraclass feature compactness. Specifically, for the *k*-th client, given a mini-batch of size *B*, the local joint loss function is defined as in Equation (9):

(9) $$L_{k}=L_{CE}+\lambda L_{Center}\;=-\sum_{i=1}^{B}\log\frac{e^{W_{y_{i}}^{T}f_{i}+b_{y_{i}}}}{\sum_{j=1}^{C}e^{W_{j}^{T}f_{i}+b_{j}}}+\frac{\lambda }{2}\sum_{i=1}^{B}{∥f_{i}-c_{y_{i}}∥}_{2}^{2}$$

where $f_{i}$ denotes the deep feature vector of the *i*-th sample output by the feature extractor; $W_{y_{i}}$ and $b_{y_{i}}$ represent the specific weight vector and bias corresponding to class $y_{i}$ in the locally isolated classifier, respectively; $c_{y_{i}}$ denotes the centre vector of class $y_{i}$ in the feature space, which is synchronously updated locally alongside the network parameters; and λ is a scalar hyperparameter that balances the interclass separability and intraclass compactness.

Notably, the aforementioned local joint optimization objective does not exist in isolation; rather, it forms an effective closed-loop synergy with the hierarchical parameter decoupling architecture proposed earlier. Specifically, $L_{CE}$ primarily drives the locally isolated classifier $\phi_{k}$ to fit the unique local label space, while the strong clustering constraint imposed by $L_{Center}$ forces the low-rank adapter to more acutely capture and offset domain-specific physical interference noise. In this way, the disentangled feature space enables the subsequently extracted public corrector $B^{\left(k\right)}$ to more purely reflect the geometric manifold characteristics of that domain, thereby providing a high-quality mathematical foundation for the alignment of the “subspace signatures” in the second phase.

### 3.6. Complexity Analysis

This section provides a theoretical evaluation of the proposed FedHSFV framework in terms of the both computational and communication complexity, demonstrating its applicability in practical federated deployment environments.

Computational Complexity: In the practical deployment of federated learning, computational latency is a crucial metric for evaluating algorithm usability. The manifold metric computation introduced in this paper was executed exclusively on the central server and exhibited high theoretical computational efficiency. Specifically, the time complexity for performing thin QR decomposition on the low-rank matrix $B^{\left(k\right)}$ is merely $O(d_{out}r^{2})$, and the subsequent SVD computation on the r×r covariance matrix requires only $O(r^{3})$ operations. In our finger vein recognition network, the output dimension of the network layers is typically in the hundreds, while the rank constraint is set to a small value (r=8). Under this configuration, the floating point operations (FLOPs) required for the aforementioned matrix factorizations have a magnitude of $10^{4}$. We conducted empirical measurements on a central server equipped with an Intel Core i9 CPU and an NVIDIA RTX 4050 GPU, and the results demonstrate that the average single-round runtime overhead for executing the aforementioned manifold metrics and aggregation weights across all clients is a mere 2.1 milliseconds. Compared with the massive computational overhead of the several GFLOPs required for the forwards and backwards propagation of deep CNN backbones on the client side as well as the hundreds of milliseconds of network transmission latency in wide-area networks, the additional computational latency introduced by this lightweight matrix decomposition on the server is practically negligible. Consequently, FedHSFV enables highly efficient, real-time personalized aggregation in practical cross-institutional deployments, breaking the computational overload bottlenecks associated with traditional high-dimensional matrix alignment.

Communication Complexity: Through the macrolevel classifier isolation strategy, this framework thoroughly circumvents the network transmission of the massive classifier parameters $\phi_{k}$; concurrently, the microlevel decoupling strategy ensures that only the extremely small low-rank signature matrix $B^{\left(k\right)}$ needs to be uploaded during the second phase. Therefore, the single-round communication volume of this framework is strictly compressed to $O(|W_{global}|+|B|)$. Specifically, the actual data volume for a single round of communication in our framework is approximately 8.9 MB. Because |B| constitutes a compact proportion of the total number of model parameters, its single-round transmission burden is almost on par with that of traditional baseline methods. More importantly, when evaluating the end-to-end total bandwidth cost, the convergence efficiency of the model must be comprehensively considered. Benefiting from the Grassmann manifold-based subspace synergy mechanism in the second phase, which can efficiently and precisely align the cross-domain optimization paths, this framework significantly accelerates the global consensus process. Empirical observations indicate that FedHSFV typically requires only approximately 20 global communication rounds to achieve stable convergence. Although the synergy of the full-rank feature extractor backbone $|W_{global}|$ is retained, because $\left|B|\ll \right|W_{global}|$ and the synchronization of the local classifier $|\phi_{k}|$ is exempted, FedHSFV achieves high-precision personalized representations while strictly guaranteeing that its communication overhead upper bound does not exceed that of traditional federated baseline methods.

## 4. Experiment

### 4.1. Datasets

To comprehensively evaluate the algorithm’s performance in real-world heterogeneous environments, in this work, selects six finger vein datasets were selected as client nodes for federated learning: SDUMLA-HMT [40], MMCBNU-6000 [41], SCUT-SFVD [42], NUPT-FV [43], VERA [44], and UTFVP [45]. Notably, since the original SCUT-SFVD database comprises both genuine samples and spoofing artefacts intended for presentation attack detection, only the genuine finger vein subset was utilized as the local client data. These datasets were acquired by different institutions on acquisition devices with varying imaging principles, encompassing diverse scenarios ranging from low to high resolution and from controlled illumination to complex backgrounds. Inherently, they constitute the Non-IID characteristics that are typical in federated learning. Detailed statistical information is summarized in Table 1.

### 4.2. Evaluation Metrics

Finger vein recognition is inherently an open-set verification task. To comprehensively and objectively quantify the model’s discriminative performance in cross-domain verification tasks, we adopted two core performance evaluation metrics that are widely used in biometrics: the equal error rate (EER) and the true acceptance rate at a specific false acceptance rate (TAR@FAR) [46].

The EER is a universal benchmark metric for measuring the comprehensive performance of a biometric authentication system. It is strictly defined as the operating point value where the false acceptance rate (FAR) equals the false rejection rate (FRR). A lower EER indicates superior overall generalization performance in terms of balancing system security and convenience. The mathematical definitions of the two fundamental error rates, the FAR and FRR, which determine the EER, are expressed in Equation (10) and Equation (11), respectively:

(10) $$FAR\left(\tau \right)=\frac{N_{FA}\left(\tau \right)}{N_{I}}$$

(11) $$FRR\left(\tau \right)=\frac{N_{FR}\left(\tau \right)}{N_{G}}$$

where τ is the given decision threshold and $N_{FA}\left(\tau \right)$ and $N_{FR}\left(\tau \right)$ represent the number of false acceptances and false rejections under this threshold, respectively. $N_{I}$ denotes the total number of interclass matching pairs (impostors), and $N_{G}$ denotes the total number of intraclass matching pairs (genuines).

Given that finger vein recognition is typically deployed in practical scenarios with stringent security requirements, such as financial payments and high-security access control, the system’s tolerance for “false acceptances” is extremely low. Therefore, relying solely on the comprehensive balance point (EER) is insufficient in terms of fully evaluating system reliability. It is thus imperative to further quantify the model’s usability under rigorous security constraints. Consequently, we introduce the TAR@FAR metric, which focuses specifically on the system’s true acceptance rate under an extremely low false acceptance rate (set to FAR = 0.01 in this paper). The TAR is defined in Equation (12):

(12) TAR=1−FRR

This metric astutely reflects the model’s ability to correctly recognize legitimate users while strictly restricting illegal intrusions. Under this setting, a higher TAR@FAR = 0.01 value signifies superior model performance.

### 4.3. Experimental Results and Analysis

To systematically evaluate the effectiveness and robustness of the FedHSFV framework in cross-device finger vein recognition tasks, we conducted progressive comparative experiments on six heterogeneous datasets. Experiment 1 verified the necessity of introducing a federated collaborative mechanism in cross-domain “data silo” scenarios by comparing it with isolated training, Experiment 2 was an ablation study that independently evaluated the enhancement of the cross-domain feature generalization capability introduced by the hierarchical decoupling mechanism, Experiment 3 verified the personalized gains derived from the subspace synergy based on the Grassmann manifold, and Experiment 4 was a comprehensive performance benchmark comparing our framework with current mainstream and state-of-the-art federated learning algorithms. Finally, in Experiment 5, a sensitivity analysis was conducted on the core hyperparameters to further demonstrate the model’s robustness and stability within specific parameter ranges. To improve the reliability of the results, we conducted the experiment three times with different random seeds. The final outcomes are reported as the mean and standard deviation across these three runs.

#### 4.3.1. Experiment 1: Comparison Between FedHSFV and Isolated Client Training

**Figure 3:**
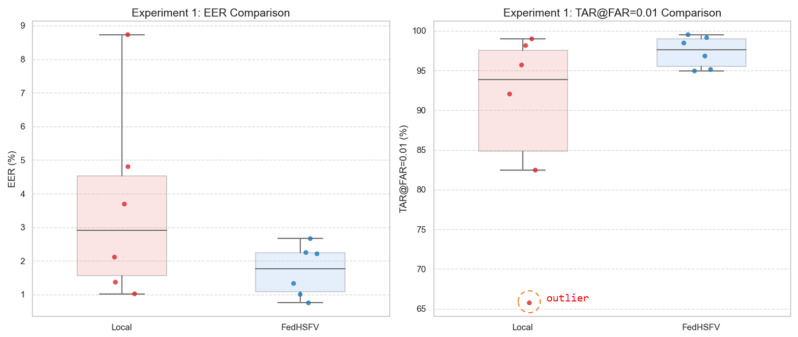
Experiment 1: Box plots of the performance distributions comparing local training and FedHSFV.

This experiment aims to verify the ability of the proposed FedHSFV framework to break “data silos” while overcoming heterogeneity obstacles. Thus, we compared it with isolated local training. In this experiment, “Local” denotes that the model was independently training by clients without mutual communication, whereas “FedHSFV” represents our proposed method. The specific EER and TAR metric results of the different training modes on each dataset are presented in Table 2, and Figure 3 intuitively visualizes the performance distribution comparison via box plots.

As observed from the quantitative data in Table 2 and Figure 3, constrained by the scarcity of the local data volume and the singularity of the distributions, the Local mode exhibits significant generalization bottlenecks. Its average EER across the six datasets reaches 3.621%, and the TAR under the stringent FAR = 0.01 threshold is merely 88.83%. Notably, on the VERA dataset, which features extremely small sample sizes, the Local model falls into severe overfitting, with the EER increasing to 8.730% and the TAR decreasing to 65.74%. In contrast, the proposed FedHSFV effectively overcomes the sample limitations of single nodes. The experiments demonstrate that the framework achieves comprehensive performance improvements across all testing nodes: the system’s average EER converges significantly to 1.701%, and the average TAR improves to 97.32%. Particularly on the most challenging dataset, VERA, benefiting from the hierarchical decoupling and subspace metric strategies, the model successfully strips away domain-specific noise and integrates knowledge from similar domains, significantly reducing the EER to 2.662% and increasing the TAR to 94.93%. In summary, FedHSFV not only successfully resolves the “data silo” dilemma but also exhibits strong robustness under rigorous verification standards.

#### 4.3.2. Experiment 2: Effectiveness Evaluation of First-Phase Hierarchical Decoupling

This experiment verifies the effectiveness of the “hierarchical parameter decoupling” mechanism in the first phase. To this end, we adopted the classic algorithm FedPer [47] as the comparative baseline. FedPer represents the traditional single-layer macroscopic decoupling paradigm that only isolates the local classifier. By comparing the complete first-phase model constructed in this paper against FedPer, we verified that the dual-dimensional hierarchical decoupling architecture is significantly superior to traditional single-layer decoupling in terms of comprehensively addressing dual data heterogeneity and extracting high-purity global consensus features.

**Figure 4:**
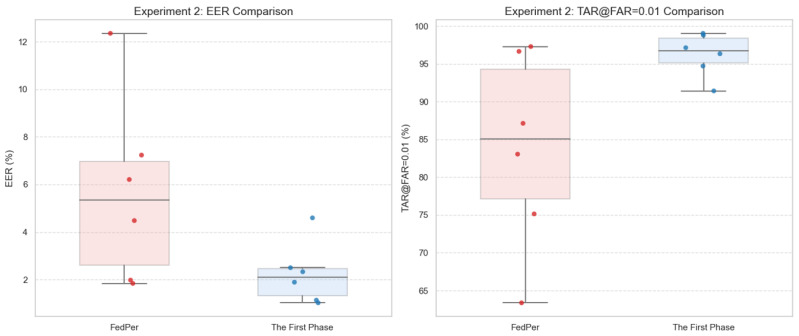
Experiment 2: Box plots comparing FedPer and The first phase of the proposed method.

Combining the quantitative data in Table 3 with Figure 4, it can be observed that the traditional serial decoupling method, FedPer, exhibits significant performance fluctuations in highly heterogeneous environments, with an average EER of 5.682%. Especially on the small-sample VERA dataset, FedPer’s EER reaches 12.349%, indicating that simple macroscopic layering struggles to effectively filter deep device-specific physical noise. Conversely, our first-phase model demonstrates strong and stable generalization capabilities. By further microscopically decomposing the feature extractor into a “global full-rank basis” bearing the consensus and a “local low-rank adapter” that captures domain shift, the model effectively separates universal vein textures from domain-specific noise. Under this mechanism, the EER on VERA is significantly reduced to 4.596%, and the average EER across the six datasets is optimized to 2.247%. The stable distribution shown in the box plots intuitively validates the robust resilience of the additive decomposition strategy when handling Non-IID finger vein data.

#### 4.3.3. Experiment 3: Effectiveness Evaluation of the Second-Phase Subspace Synergy

**Figure 5:**
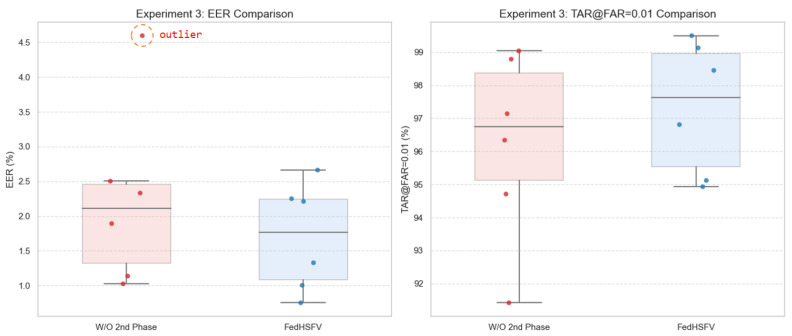
Experiment 3: Box plots comparing the proposed method W/O the 2nd phase and the complete FedHSFV.

This experiment further validated the effectiveness of the second phase in the FedHSFV framework. By comparing the performance difference between executing solely the first phase and executing the complete two-stage framework, we effectively evaluated the critical role of the Grassmann manifold-based weighted aggregation mechanism in terms of addressing data heterogeneity. The specific performance comparison metrics for this ablation study are shown in Table 4, and Figure 5 presents box plots that compare the performance fluctuations with and without the introduction of the second phase.

The data in Table 4 and the corresponding box plots reveal a consistent trend of performance improvement. Upon introducing the second-phase subspace synergy, the recognition accuracy across all clients achieves a secondary enhancement: the overall average EER further converges from 2.248% to 1.703%, and the average TAR@FAR = 0.01 under high-security thresholds improves from 96.24% to 97.32%.

This performance increase caused by “precise synergy” is most vividly demonstrated on edge nodes with scarce data. Taking the VERA dataset as an example, the weighted aggregation in the second phase significantly reduces its EER from 4.596% to 2.662% and increases its TAR@FAR = 0.01 by 1.08%. This significant improvement effectively demonstrates that discarding the traditional Euclidean distance in the parameter space in favour of measuring the geometric alignment of the subspaces on the manifold space can more accurately characterize the true distribution similarity among heterogeneous clients. This mechanism successfully guides the central server to generate optimal personalized aggregation weights, enabling data-scarce nodes to securely assimilate positive knowledge from nodes with similar feature distributions while effectively suppressing interference from irrelevant domains.

#### 4.3.4. Experiment 4: Comparison with State-of-the-Art Algorithms

**Figure 6:**
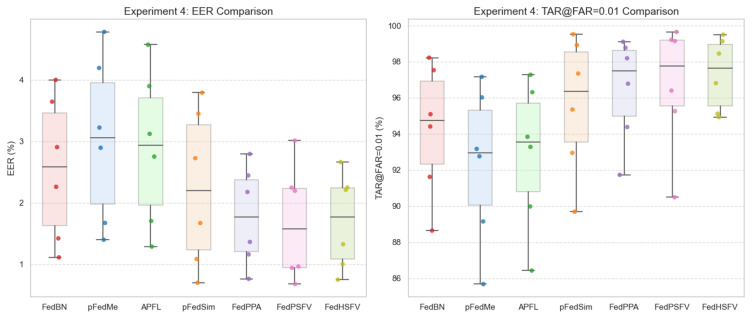
Experiment 4: Box plots comparing the SOTA algorithms.

In this experiment, we benchmarked FedHSFV against existing mainstream and state-of-the-art personalized federated learning algorithms, including FedBN [48], which retains Batch Normalization layers locally without participating in server aggregation; pFedMe [49], which utilizes Moreau Envelopes as a regularization term; the hybrid personalized federated learning approach APFL [50]; the similarity-based PFL algorithm pFedSim [18]; the progressive parameter alignment-based FedPPA [19]; and the prototype knowledge sharing-based FedPSFV [35]. All of these approaches are currently employed in the finger vein domain. The specific recognition performance of each method across the six datasets is detailed in Table 5, and Figure 6 provides the corresponding box plot distributions, where the dashed lines inside the boxes represent the mean values.

In terms of the overall recognition performance, FedHSFV demonstrates significant cross-domain generalization advantages, achieving the highest average TAR@FAR = 0.01 across the board at 97.32%. Furthermore, the average EER of our framework (1.701%) significantly outperforms FedBN (2.557%), pFedMe (3.027%), APFL (2.889%), pFedSim (2.238%), and FedPPA (1.785%) and is only marginally higher than FedPSFV (1.674%).

Although the average EER of FedHSFV is slightly higher than that of FedPSFV, this difference stems primarily from the local performance offsets generated by the baseline algorithm on certain simple data distributions. When evaluating the critical metric of performance, the lower bounds, which reflect federated learning’s ability to overcome extreme data heterogeneity, FedHSFV has a clear advantage. Taking the VERA dataset, which has the most complex distribution and scarce samples, as an example, FedPSFV experiences severe generalization degradation (the EER increases to 3.011%, and the TAR decreases to 90.49%), whereas FedHSFV successfully maintains the lowest EER of 2.662% and the highest TAR of 94.93%. This finding thoroughly validates that our framework possesses superior antidegradation capabilities and heterogeneous domain adaptability when confronted with extreme Non-IID data.

To further quantify the global stability of the algorithms in cross-domain deployments, Table 6 compares the EER statistical distributions of our framework and FedPSFV.

As illustrated in Table 6, both the sample variance and the standard deviation of the EER for FedHSFV are lower than those for FedPSFV. This finding indicates that, due to the precise directional synergy guided by the subspace metric, FedHSFV effectively evades the risk of local domain overfitting. At the cost of a marginal average precision trade-off, it achieves minimization of the cross-domain recognition accuracy variance. After Synthesizing all of the benchmark comparison results, FedHSFV not only leads the existing SOTA algorithms in terms of TAR@FAR = 0.01 but also exhibits prominent advantages in terms of overcoming extreme heterogeneity and safeguarding cross-domain stability, thereby providing a highly reliable and robust personalized federated learning paradigm for practical cross-institutional finger vein recognition deployments.

#### 4.3.5. Experiment 5: Hyperparameter Sensitivity Analysis

In the first four core comparative experiments, all of the hyperparameters of our framework adhered to a unified, empirically optimal configuration (r=8, τ=0.1). To provide a deeper exploration of the robustness of the FedHSFV framework under varying parameter configurations and to verify that its outstanding performance gains do not stem merely from specific parameter tuning, this section describes a comprehensive sensitivity analysis that was conducted on these two core hyperparameters to evaluate the model’s performance stability under parameter perturbations.

A. Impact of the Low-Rank Constraint Dimension r

**Figure 7:**
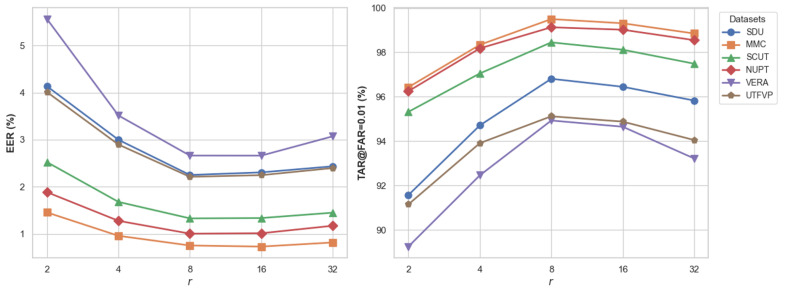
Impact of the rank on the EER and TAR@FAR = 0.01.

Table 7 and Table 8 show the impact of different *r* values on the EER and TAR@FAR = 0.01, respectively, and Figure 7 presents the corresponding line charts.

In the microscopic additive decoupling mechanism, the parameter *r* determines the representational capacity of the local low-rank adapter, thus playing a decisive role in isolating device-specific physical noise. As illustrated in the charts, when *r* is assigned a smaller value (r=2), the excessively narrow information bottleneck of the adapter hinders the model from fully capturing complex local domain shifts. Consequently, some specific noise remains coupled with the global features, leading to certain performance fluctuations. However, when *r* varies within the broad interval of [8,16], the model’s EER and TAR across all datasets remain consistently low and stable. This finding indicates that without deliberately seeking a specific rank setting, as long as the model is granted an appropriate low-rank space, the microscopic additive decomposition can exhibit outstanding and stable feature decoupling capabilities. Although further increasing *r* (r=32) causes slight overfitting and performance degradation on limited local samples due to the introduction of redundant parameters and the disruption of the low-rank manifold assumption, overall, this mechanism demonstrates strong robustness to the selection of *r*.

B. Impact of the Temperature Coefficient τ

**Figure 8:**
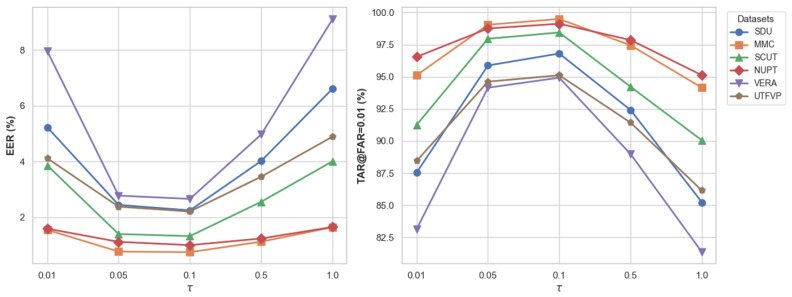
Impact of the temperature on EER and TAR@FAR = 0.01.

Table 9 and Table 10 illustrate the impact of different τ values on the EER and TAR@FAR = 0.01, respectively. The corresponding line charts are shown in Figure 8.

During the personalized synergy phase, which is based on the subspace metric, the temperature coefficient τ plays a critical role in regulating the “sharpness” of the similarity weight distribution. As depicted in the charts, within the interval of τ∈[0.05,0.1], our framework consistently maintains excellent and stable recognition accuracy. This finding demonstrates that the subspace collaborative mechanism can effectively filter heterogeneous noise and achieve precise positive knowledge transfer across a relatively wide parameter domain. Analysing extreme boundary cases, as τ approaches a larger value (τ=1.0), the weight distribution tends towards uniformity, and the algorithm’s aggregation behaviour degrades into indiscriminative knowledge fusion akin to traditional FedAvg, thereby weakening its capacity to resist negative transfer. Conversely, when τ is set to an extremely small value (τ=0.01), the weight distribution collapses into an approximate one-hot vector, causing the clients to overly rely on a single highly weighted correlated node that is prone to trapping the model in local optima. However, in practical deployments, as long as these extreme values are avoided, the model can consistently exhibit commendable performance.

## 5. Conclusions

To address the pervasive challenge of data heterogeneity in cross-domain federated deployments, this paper proposes a novel personalized federated learning framework, termed FedHSFV. Unlike the existing paradigms, which predominantly rely on local data for personalized fine-tuning, this study proposes a hierarchical parameter decoupling architecture. Macroscopically, the classifier is explicitly isolated at the local level to avert dimensional conflicts; microscopically, an additive parameter decomposition is introduced into the feature extractor. This dual-level design effectively disentangles domain-invariant vein semantics from device-specific physical noise while maintaining high communication efficiency. Furthermore, we depart from traditional Euclidean distance metrics in the parameter space, opting instead to utilize principal angles on the Grassmann manifold to quantify the subspace similarities among clients. This geometric perspective not only overcomes the inherent rotational ambiguity of neural network parameters but also provides a more robust “ally” identification mechanism for personalized collaborative aggregation. The experimental results demonstrate that FedHSFV exhibits significant advantages in terms of both enhancing the overall recognition performance and mitigating the performance degradation induced by data heterogeneity.

## Figures and Tables

**Table 1:** Statistical information of the six finger vein datasets.

Datasets	Categories	Total Images	Training Set	Test Set
SDU	636	3816	3054	976
MMC	600	6000	4800	1200
SCUT	101	3636	2916	720
NUPT	1680	16,800	13,440	3360
VERA	220	440	352	88
UTFVP	360	1440	1152	288

**Table 2:** Performance comparison between local training and FedHSFV.

Datasets	Local		FedHSFV	
	**EER (%)**	**TAR@FAR = 0.01 (%)**	**EER (%)**	**TAR@FAR = 0.01 (%)**
SDU	3.691 ± 0.014	92.03 ± 0.12	2.250 ± 0.008	96.81 ± 0.09
MMC	1.367 ± 0.011	98.13 ± 0.11	0.752 ± 0.010	99.50 ± 0.09
SCUT	2.112 ± 0.013	95.68 ± 0.08	1.328 ± 0.008	98.45 ± 0.11
NUPT	1.021 ± 0.011	98.97 ± 0.08	1.004 ± 0.007	99.13 ± 0.10
VERA	8.730 ± 0.018	65.74 ± 0.16	2.662 ± 0.011	94.93 ± 0.12
UTFVP	4.804 ± 0.016	82.45 ± 0.12	2.211 ± 0.012	95.12 ± 0.12
Best	1.021 ± 0.009	98.97 ± 0.08	0.752 ± 0.010	99.50 ± 0.09
Worse	8.730 ± 0.017	65.74 ± 0.14	2.662 ± 0.011	94.93 ± 0.12
Average	3.621 ± 0.014	88.83 ± 0.11	1.701 ± 0.009	97.32 ± 0.11

**Table 3:** Effectiveness evaluation of the first-phase hierarchical decoupling.

Datasets	FedPer		The First Phase	
	**EER (%)**	**TAR@FAR = 0.01 (%)**	**EER (%)**	**TAR@FAR = 0.01 (%)**
SDU	7.233 ± 0.013	83.05 ± 0.16	2.331 ± 0.009	96.34 ± 0.10
MMC	1.840 ± 0.006	96.65 ± 0.09	1.025 ± 0.006	99.04 ± 0.08
SCUT	4.481 ± 0.010	87.13 ± 0.13	1.892 ± 0.008	97.14 ± 0.09
NUPT	1.980 ± 0.007	97.29 ± 0.08	1.137 ± 0.007	98.79 ± 0.08
VERA	12.349 ± 0.019	63.37 ± 0.22	4.596 ± 0.013	91.42 ± 0.14
UTFVP	6.208 ± 0.012	75.14 ± 0.17	2.502 ± 0.011	94.71 ± 0.11
Best	1.841 ± 0.006	97.29 ± 0.08	1.025 ± 0.006	99.04 ± 0.08
Worse	12.349 ± 0.019	63.37 ± 0.22	4.596 ± 0.013	91.42 ± 0.14
Average	5.682 ± 0.011	83.77 ± 0.14	2.247 ± 0.009	96.24 ± 0.10

**Table 4:** Effectiveness evaluation of the second-phase subspace synergy.

Datasets	W/O 2nd Phase		FedHSFV	
	**EER (%)**	**TAR@FAR = 0.01 (%)**	**EER (%)**	**TAR@FAR = 0.01 (%)**
SDU	2.331 ± 0.009	96.34 ± 0.10	2.250 ± 0.008	96.81 ± 0.09
MMC	1.025 ± 0.006	99.04 ± 0.08	0.752 ± 0.010	99.50 ± 0.09
SCUT	1.892 ± 0.008	97.14 ± 0.09	1.328 ± 0.008	98.45 ± 0.11
NUPT	1.137 ± 0.007	98.79 ± 0.08	1.004 ± 0.007	99.13 ± 0.10
VERA	4.596 ± 0.013	91.42 ± 0.14	2.662 ± 0.011	94.93 ± 0.12
UTFVP	2.502 ± 0.011	94.71 ± 0.11	2.211 ± 0.012	95.12 ± 0.12
Best	1.025 ± 0.006	99.04 ± 0.08	0.752 ± 0.010	99.50 ± 0.09
Worse	4.596 ± 0.013	91.42 ± 0.14	2.662 ± 0.011	94.93 ± 0.12
Average	2.247 ± 0.009	96.24 ± 0.10	1.701 ± 0.009	97.32 ± 0.11

**Table 5:** Comprehensive performance comparison with SOTA methods.

Datasets	FedBN		pFedMe		APFL		pFedSim		FedPPA		FedPSFV		FedHSFV	
	**EER (%)**	**TAR@FAR = 0.01 (%)**	**EER (%)**	**TAR@FAR = 0.01 (%)**	**EER (%)**	**TAR@FAR = 0.01 (%)**	**EER (%)**	**TAR@FAR = 0.01 (%)**	**EER (%)**	**TAR@FAR = 0.01 (%)**	**EER (%)**	**TAR@FAR = 0.01 (%)**	**EER (%)**	**TAR@FAR = 0.01 (%)**
SDU	2.905 ± 0.009	94.41 ± 0.10	3.223 ± 0.010	93.17 ± 0.08	3.121 ± 0.012	93.84 ± 0.08	2.726 ± 0.009	95.35 ± 0.11	2.177 ± 0.009	96.78 ± 0.09	2.248 ± 0.007	96.40 ± 0.08	2.250 ± 0.008	96.81 ± 0.09
MMC	1.114 ± 0.007	98.22 ± 0.08	1.401 ± 0.013	97.16 ± 0.10	1.289 ± 0.013	97.27 ± 0.10	0.702 ± 0.010	99.52 ± 0.09	0.765 ± 0.007	99.10 ± 0.11	0.680 ± 0.010	99.65 ± 0.08	0.752 ± 0.010	99.50 ± 0.09
SCUT	2.261 ± 0.010	95.09 ± 0.11	2.895 ± 0.012	92.76 ± 0.08	2.752 ± 0.013	93.28 ± 0.10	1.673 ± 0.012	97.34 ± 0.11	1.365 ± 0.008	98.19 ± 0.13	0.967 ± 0.009	99.21 ± 0.11	1.328 ± 0.008	98.45 ± 0.11
NUPT	1.423 ± 0.009	97.53 ± 0.09	1.674 ± 0.011	96.02 ± 0.10	1.705 ± 0.013	96.31 ± 0.09	1.087 ± 0.008	98.92 ± 0.10	1.163 ± 0.005	98.77 ± 0.08	0.944 ± 0.007	99.14 ± 0.10	1.004 ± 0.007	99.13 ± 0.10
VERA	3.998 ± 0.016	88.64 ± 0.13	4.778 ± 0.015	85.68 ± 0.12	4.570 ± 0.016	86.43 ± 0.14	3.790 ± 0.011	89.69 ± 0.13	2.794 ± 0.014	91.73 ± 0.12	3.011 ± 0.013	90.49 ± 0.13	2.662 ± 0.011	94.93 ± 0.12
UTFVP	3.644 ± 0.014	91.62 ± 0.14	4.192 ± 0.013	89.15 ± 0.10	3.897 ± 0.015	89.98 ± 0.14	3.450 ± 0.012	92.95 ± 0.15	2.445 ± 0.009	94.38 ± 0.13	2.196 ± 0.012	95.26 ± 0.13	2.211 ± 0.012	95.12 ± 0.12
Best	1.114 ± 0.009	98.22 ± 0.08	1.401 ± 0.013	97.16 ± 0.10	1.289 ± 0.013	97.27 ± 0.10	0.702 ± 0.010	99.52 ± 0.09	0.765 ± 0.007	99.10 ± 0.11	0.680 ± 0.010	99.65 ± 0.08	0.752 ± 0.010	99.50 ± 0.09
Worse	3.998 ± 0.016	88.64 ± 0.13	4.778 ± 0.015	85.68 ± 0.12	4.570 ± 0.016	86.43 ± 0.14	3.790 ± 0.011	89.69 ± 0.13	2.794 ± 0.014	91.73 ± 0.12	3.011 ± 0.013	90.49 ± 0.13	2.662 ± 0.011	94.93 ± 0.12
Average	2.557 ± 0.012	94.25 ± 0.11	3.027 ± 0.013	92.32 ± 0.10	2.889 ± 0.013	92.85 ± 0.11	2.238 ± 0.010	95.63 ± 0.12	1.785 ± 0.009	96.49 ± 0.09	1.674 ± 0.010	96.69 ± 0.11	1.701 ± 0.009	97.32 ± 0.11

**Table 6:** Statistical Distribution of EER for FedPSFV and FedHSFV.

Method	Average EER	EER Variance	EER Std. Dev.
FedPSFV	1.674%	0.735	0.857
FedHSFV	1.701%	0.502	0.708

**Table 7:** Impact of the rank *r* on the EER (%).

*r*	SDU	MMC	SCUT	NUPT	VERA	UTFVP
2	4.133 ± 0.015	1.454 ± 0.011	2.522 ± 0.014	1.884 ± 0.012	5.561 ± 0.018	4.010 ± 0.016
4	2.997 ± 0.012	0.958 ± 0.008	1.678 ± 0.010	1.276 ± 0.009	3.513 ± 0.014	2.896 ± 0.013
8	2.250 ± 0.008	0.752 ± 0.010	1.328 ± 0.008	1.004 ± 0.007	2.662 ± 0.011	2.211 ± 0.012
16	2.305 ± 0.009	0.729 ± 0.011	1.335 ± 0.009	1.012 ± 0.008	2.661 ± 0.012	2.246 ± 0.011
32	2.434 ± 0.011	0.816 ± 0.012	1.449 ± 0.010	1.171 ± 0.009	3.071 ± 0.013	2.397 ± 0.012

**Table 8:** Impact of the rank *r* on the TAR@FAR = 0.01 (%).

*r*	SDU	MMC	SCUT	NUPT	VERA	UTFVP
2	91.57 ± 0.22	96.43 ± 0.15	95.32 ± 0.18	96.25 ± 0.16	89.25 ± 0.25	91.15 ± 0.21
4	94.71 ± 0.14	98.34 ± 0.11	97.05 ± 0.13	98.18 ± 0.12	92.46 ± 0.17	93.91 ± 0.15
8	96.81 ± 0.09	99.50 ± 0.09	98.45 ± 0.11	99.13 ± 0.10	94.93 ± 0.12	95.12 ± 0.12
16	96.45 ± 0.11	99.31 ± 0.10	98.12 ± 0.12	99.02 ± 0.11	94.65 ± 0.14	94.88 ± 0.13
32	95.83 ± 0.15	98.85 ± 0.13	97.49 ± 0.14	98.55 ± 0.13	93.22 ± 0.18	94.05 ± 0.16

**Table 9:** Impact of the temperature Coefficient τ on EER (%).

τ	SDU	MMC	SCUT	NUPT	VERA	UTFVP
0.01	5.231 ± 0.017	1.551 ± 0.012	3.864 ± 0.015	1.595 ± 0.011	7.964 ± 0.021	4.119 ± 0.016
0.05	2.448 ± 0.010	0.774 ± 0.011	1.402 ± 0.009	1.122 ± 0.008	2.779 ± 0.012	2.383 ± 0.013
0.1	2.250 ± 0.008	0.752 ± 0.010	1.328 ± 0.008	1.004 ± 0.007	2.662 ± 0.011	2.211 ± 0.012
0.5	4.025 ± 0.015	1.128 ± 0.010	2.560 ± 0.013	1.242 ± 0.009	4.977 ± 0.017	3.461 ± 0.014
1.0	6.619 ± 0.019	1.647 ± 0.013	4.015 ± 0.016	1.655 ± 0.012	9.114 ± 0.024	4.896 ± 0.018

**Table 10:** Impact of the temperature coefficient τ on TAR@FAR = 0.01 (%).

τ	SDU	MMC	SCUT	NUPT	VERA	UTFVP
0.01	87.54 ± 0.25	95.12 ± 0.17	91.25 ± 0.21	96.55 ± 0.16	83.13 ± 0.31	88.45 ± 0.24
0.05	95.88 ± 0.12	99.05 ± 0.10	97.96 ± 0.13	98.76 ± 0.11	94.15 ± 0.15	94.62 ± 0.14
0.1	96.81 ± 0.09	99.50 ± 0.09	98.45 ± 0.11	99.13 ± 0.10	94.93 ± 0.12	95.12 ± 0.12
0.5	92.41 ± 0.18	97.45 ± 0.13	94.22 ± 0.16	97.86 ± 0.14	88.98 ± 0.22	91.43 ± 0.19
1.0	85.22 ± 0.28	94.15 ± 0.19	90.05 ± 0.23	95.12 ± 0.18	81.35 ± 0.35	86.14 ± 0.27

## Data Availability

The data presented in this study are publicly available in the SDUMLA-HMT [40], MMCBNU-6000 [41], SCUT-SFVD [42], NUPT-FV [43], VERA [44], and UTFVP [45] datasets.
